# Supplementary material for: Pulmonary complications observed in patients with infective endocarditis with and without injection drug use: An analysis of the National Inpatient Sample
Source: PLoS One. 2021 Sep 3;16(9):e0256757. doi: 10.1371/journal.pone.0256757 (PMC8415585; doi:10.1371/journal.pone.0256757)
Supplement: S1 File — (DOCX) [file pone.0256757.s001.docx]

**S1 File**

**Supplement Tables**

*S1 Table.* **ICD-10-CM Diagnosis Codes**

*S2 Table.* **ICD-10-CM Procedural Codes (Abbreviated)**

*S3 Table.* **Association between pulmonary complications, compared to no pulmonary complications, on patient demographics and hospital characteristics**

*S4 Table.* **Odds ratios and regression results for univariate model of outcomes and hospital region**

S5 *Table*. **Adjusted association of pulmonary complications, compared to no**

**complication, on inpatient outcomes, stratified by drug use.**

**S1 Table. ICD-10-CM Diagnosis Codes**

|  | **ICD-10-CM** |
| --- | --- |
| **Infective Endocarditis** | A32.82 |
|  | B37.6 |
|  | I33.0 |
|  | I33.9 |
|  | I38 |
|  | I39 |
| **Septic Pulmonary Embolism** | I26.01 |
|  | I26.90 |
| **Lung Abscess** | J85.0 |
|  | J85.1 |
|  | J85.2 |
|  | J86.0 |
| **Empyema** | J86.9 |
|  | F11.1 |
| **Intracranial Abscess/**  **Granuloma** | G06.0 |
| **Intracerebral Hemorrhage** | I60.0X |
|  | I60.1X |
|  | I60.2 |
|  | I60.3X |
|  | I60.4 |
|  | I60.5X |
|  | I60.6-I60.9 |
|  | I61.X-I62.X |
| **Cerebral Infarction** | 163.X |
| **Cerebral Ischemia** | I67.82 |
| **Opiates/Opioid** | F11.2 |
|  | F11.9 |
|  | T40.0X1A –  T40.0X6S |
|  | T40.1X1A –  T40.1X4S |
|  | T40.2X1A –  T40.2X6S |
|  | T40.3X1A –  T40.3X6S |
|  | T40.4X1A –  T40.3X6S |
|  | T40.60 |
|  | T40.69 |
|  | Y45.0 |
|  | T42.3X1A – T42.3X6S |
| **Benzodiazepines** | T42.4X1A –  T42.4X6S |
|  | F14.1 |
| **Cocaine** | F14.2 |
|  | F14.9 |
|  | T40.5X1A –  T40.5X6S |
|  | F15.1 |
| **Amphetamines or Stimulants** | F15.2 |
|  | F15.9 |
|  | T43.62 |
|  | T43.63 |
|  | T43.60 |
|  | T43.69 |
|  | F16.1 |
| **Hallucinogens** | F16.2 |
|  | F16.9 |
|  | T40.8X1A –  T40.8X4S |
|  | T40.90 |
|  | T40.99 |
|  | F13.1 |
| **Other Drugs** | F13.2 |
|  | F13.9 |
|  | F19.1 |
|  | F19.2 |
|  | F19.9 |
|  | T42.6X1A –  T42.6X6S |
|  | T42.7 |
|  | T41.1X1A –  T41.1X6S |
|  | T43.50 |
|  | T43.59 |
|  | T43.8X1A –  T42.8X6S |
|  | T43.9 |
|  | X42 |
|  | X62 |
|  | Y12 |
|  | B17.10 |
| **Hepatitis C** | B17.11 |
|  | B18.2 |
|  | B19.20 |
|  | B19.21 |
|  | Z22.52 |

**S2 Table. ICD-10-CM Procedural Codes (Abbreviated)**

|  | **ICD-10-CM** |
| --- | --- |
| **Heart Valve Surgery** | 024F |
|  | 024G |
|  | 024J |
|  | 025F |
|  | 025G |
|  | 025H |
|  | 025J |
|  | 027F |
|  | 027G |
|  | 027H |
|  | 027J |
|  | 02BF |
|  | 02BG |
|  | 02BH |
|  | 02BJ |
|  | 02CF |
|  | 02CG |
|  | 02CH |
|  | 02CJ |
|  | 02LH |
|  | 02NF |
|  | 02NG |
|  | 02NH |
|  | 02NJ |
|  | 02QF |
|  | 02QG |
|  | 02QH |
|  | 02QJ |
|  | 02RF |
|  | 02RG |
|  | 02RH |
|  | 02RJ |
|  | 02TH |
|  | 02UF |
|  | 02UG |
|  | 02UH |
|  | 02UJ |
|  | 02VG |
|  | 02WF |
|  | 02WG |
|  | 02WH |
|  | 02WJ |
|  | X2RF032 |
|  | X2RF332 |
|  | X2RF432 |
| **Lung Resection** | 0BTC |
|  | 0BTD |
|  | 0BTF |
|  | 0BTG |
|  | 0BTH |
|  | 0BTJ |
|  | 0BTK |
|  | 0BTL |
|  | 0BTM |
| **Decortication** | 0BDN |
|  | 0BDP |
|  | 0BBN |
|  | 0BBP |
|  | 0B5N |
|  | 0B5P |
| **Drainage of Pleura** | 0B9N |
|  | 0B9P |
|  | 0W99 |
|  | 0W9B |

*****For coding purposes, ICD-10 procedure codes were abbreviated to identify all codes that began with the following strings seen in this table.

**02RF, 02RJ, O2RG, 02RJ, X2RF are valve replacement procedure codes.

**S3 Table. Association between pulmonary complications, compared to no pulmonary complications, on patient demographics and hospital characteristics**

|  | **Pulmonary Complication^a^**  **6,580 (7%)** | **No Pulmonary Complication**  **82,415 (93%)** | **OR (95% CI)** | **p-value** |
| --- | --- | --- | --- | --- |
| **Age, years, median (IQR)** | 34 (27 – 46) | 66 (52 – 78) | 0.95  (0.95, 0.95) | <0.0001 |
| **Gender** |  |  |  |  |
| Male, n (%) | 3,145 (48) | 44,630 (54) | 1.0 (ref) | -- |
| Female, n (%) | 3,435 (52) | 37,785 (46) | 0.93  (0.81, 1.08) | 0.35 |
| **Race/ethnicity, n (%)** |  |  |  |  |
| Non-Hispanic White | 5,025 (81) | 57,115 (72) | 1.0 (ref) | -- |
| Non-Hispanic Black | 560 (9) | 10,725 (14) | 0.75  (0.59, 0.96) | 0.02 |
| Hispanic | 420 (7) | 7,090 (9) | 0.71  (0.54, 0.95) |  |
| Other^b^ | 195 (3) | 4,065 (5) | 0.72  (0.50, 1.03) | 0.07 |
| **Primary insurance, n (%)** |  |  |  |  |
| Medicare/Medicaid | 4,275 (66) | 63,330 (77) | 1.0 (ref) | -- |
| Private | 1,070 (17) | 13,990 (17) | 0.90  (0.74, 1.09) | 0.27 |
| Self-pay/other | 1,120 (17) | 4,690 (6) | 1.36  (1.08, 1.72) | 0.01 |
| **Median ZIP code income^c^, n (%)** |  |  |  |  |
| <$43,000 | 2,620 (41) | 26,735 (33) | 1.21  (0.96, 1.52) | 0.10 |
| $43,000-$53,999 | 1,725 (27) | 21,115 (26) | 1.13  (0.90, 1.42) | 0.31 |
| $54,000-$70,999 | 1,230 (19) | 17,630 (22) | 1.01  (0.79, 1.29) | 0.95 |
| ≥$71,000 | 875 (13) | 15,205 (19) | 1.0 (ref) | -- |
| **CCI, median (IQR)** | 0 (0 – 1) | 2 (0 – 3) | 0.84  (0.80, 0.88) | <0.0001 |
| **IV drug use, n (%)^d^** | 4,395 (67) | 11,095 (13) | 2.97  (2.50, 3.45) | <0.0001 |
| **Hospital bed size, n (%)** |  |  |  |  |
| Small | 930 (14) | 13,625 (17) | 1.03  (0.84, 1.27) | 0.77 |
| Medium | 1,625 (25) | 23,305 (28) | 0.96  (0.80, 1.15) | 0.67 |
| Large | 4,025 (61) | 45,485 (55) | 1.0 (ref) | -- |
| **Hospital location/type, n (%)** |  |  |  |  |
| Rural, non-teaching | 375 (6) | 7,575 (9) | 1.0 (ref) | -- |
| Urban, non-teaching | 1,325 (20) | 20,195 (25) | 1.48  (1.06, 2.07) | 0.02 |
| Urban, teaching | 4,880 (74) | 54,645 (66) | 1.58  (1.16, 2.15) | 0.004 |
| **Hospital region, n (%)** |  |  |  |  |
| South | 3,015 (46) | 34,670 (42) | 1.0 (ref) | -- |
| Northeast | 1,030 (16) | 15,650 (19) | 0.85  (0.67, 1.08) | 0.18 |
| Midwest | 1,325 (20) | 17,235 (21) | 1.08  (0.88, 1.32) | 0.46 |
| West | 1,210 (18) | 14,860 (18) | 1.04  (0.84, 1.30) | 0.72 |
| **Hospital ownership, n (%)** |  |  |  |  |
| Government, nonfederal | 1,000 (15) | 9,570 (12) | 1.0 (ref) | -- |
| Private, non-profit | 4,870 (74) | 59,370 (72) | 1.03  (0.84, 1.27) | 0.77 |
| Private, invest-own | 710 (11) | 13,475 (16) | 0.81  (0.60, 1.08) | 0.15 |
| **Elective admission, n (%)** | 375 (6) | 8,360 (10) | 1.46  (1.10, 1.94) | 0.01 |
| **ED services, n (%)** | 4,170 (63) | 55,990 (68) | 0.96  (0.79, 1.17) | 0.70 |
| **Admission source, n (%)** |  |  |  |  |
| Non-transfer | 4,530 (69) | 66,720 (81) | 1.0 (ref) | -- |
| Acute care transfer | 1,795 (28) | 11,680 (14) | 1.76  (1.40, 2.21) | <0.0001 |
| Other transfer | 220 (3) | 3,695 (5) | 1.26  (0.89, 1.77) | 0.19 |
| **Cerebral complication, n (%)^f^** | 480 (7) | 8,780 (11) | 0.58  (0.45, 0.76) | <0.0001 |
| Abbreviations: ED, emergency department; CCI, Charlson comorbidity index  ^a^ Complications included pyothorax, lung abscess, and septic pulmonary embolism; patients were able to have more than one complication  ^b^ Other race includes Asian/Pacific Islander, Native American, and ‘Other’ as categorized by HCUP; races were collapsed due to small cell sizes  ^c^ Quartile classification of the estimated median household income of residents in the patient's ZIP Code  ^d^ Drug use was categorized as opioid use (with or without other drugs), cocaine (with or without other non-opioid drugs), other drugs (no opioid or cocaine), and Hepatitis C only  ^d^ Other drugs include benzodiazepines, amphetamines or stimulants, and hallucinogens.  ^e^ Includes cerebral abscess, hemorrhage, infarction, and ischemia | | | | |

**S4 Table.** **Odds ratios and regression results for univariate model of outcomes and hospital region**

| **Outcomes by Hospital Region** | **OR (95% CI)** | **p-value** |
| --- | --- | --- |
| **Disposition and hospital region** |  |  |
| Routine (Northeast v. South) | 1.48 (1.31, 1.67) | <.0001 |
| Home health care (Northeast v. South) | 1.81 (1.55, 2.10) | <.0001 |
| Transferred (Northeast v. South) | 1.75 (1.57, 1.94) | <.0001 |
| Died (Northeast v. South) | 1.58 (1.35, 1.85) | <.0001 |
| Routine (Midwest v. South) | 1.14 (1.02, 1.29) | 0.0244 |
| Home health care (Midwest v. South) | 1.37 (1.18, 1.58) | <.0001 |
| Transferred (Midwest v. South) | 1.45 (1.31, 1.60) | <.0001 |
| Died (Midwest v. South) | 1.23 (1.05, 1.43) | 0.0092 |
| Routine (West v. South) | 0.85 (0.75, 0.96) | 0.0069 |
| Home health care (West v. South) | 1.05 (0.91, 1.23) | 0.4965 |
| Transferred (West v. South) | 0.93 (0.84, 1.03) | 0.1439 |
| Died (West v. South) | 1.02 (0.87, 1.19) | 0.7895 |
| **Thoracic procedure and hospital region** |  |  |
| Northeast v. South | 1.25 (1.03, 1.50) | 0.0219 |
| Midwest v. South | 1.10 (0.92, 1.30) | 0.3284 |
| West v. South | 0.95 (0.80, 1.19) | 0.532 |
| **Heart valve surgery and hospital region** |  |  |
| Northeast v. South | 1.13 (0.90, 1.41) | 0.2819 |
| Midwest v. South | 1.15 (0.84, 1.56) | 0.3859 |
| West v. South | 0.96 (0.76, 1.21) | 0.7137 |
| **Length of stay (days) and hospital region** |  | 0.0058 |
| Hospital charges (thousands) and hospital region |  | 0.0044 |
| Thoracic procedure (days) and hospital region |  | 0.0053 |
| Heart valve surgery (days) and hospital region |  | 0.0146 |
| Abbreviations: OR, odds ratio |  |  |

**S5 Table. Adjusted association of pulmonary complications, compared to no complication, on inpatient outcomes, stratified by drug use.**

|  | **DU-IE** | **Non-DU IE** |  |
| --- | --- | --- | --- |
|  | **OR (95% CI)^a^** | **OR (95% CI)^a^** | **p-value^b^** |
| **Discharge disposition** |  |  |  |
| Routine | 1.0 (ref) | 1.0 (ref) | – |
| Home health care | 1.62 (1.19, 2.21) | 0.80 (0.56, 1.14) | 0.005 |
| Transfer, acute care | 2.12 (1.46, 3.08) | 1.10 (0.85, 1.44) | 0.004 |
| Transfer, long-term care | 2.98 (2.22, 3.99) | 1.90 (1.53, 2.36) | 0.01 |
| Died | 2.56 (1.72, 3.83) | 1.48 (1.05, 2.10) | 0.04 |
| **Inpatient procedure** |  |  |  |
| Thoracic procedure | 5.52 (4.14, 7.36) | 6.81 (5.16, 8.98) | 0.27 |
| Heart valve surgery | 0.92 (0.64, 1.32) | 1.05 (0.79, 1.39) | 0.54 |
|  | **CIE (95% CI)^a^** | **CIE (95% CI)^a^** | **p-value^b^** |
| **Length of stay, days** | 7.50 (5.81, 9.20) | 7.26 (6.14, 8.38) | 0.68 |
| **Hospital charges, thousands** | 110.67 (70.46, 150.89) | 83.70 (60.30, 107.10) | 0.01 |
| **Thoracic procedure^c^, days** | 0.37 (-0.14, 0.89) | 0.69 (0.31, 1.07) | 0.21 |
| **Heart valve surgery^c^, days** | 2.10 (-0.06, 4.26) | 2.72 (1.36, 4.07) | 0.37 |
| Abbreviations: DU, drug use; IE, infection endocarditis OR, odds ratio; CI, confidence interval; IQR; interquartile range; CIE, change in estimate  ^a^ Adjusted for age, sex, race/ethnicity, primary insurance, median income in the patient’s ZIP code, Charlson Comorbidity Index (CCI), IV drug use, hospital bed size, teaching status, region, ownership, elective admission, ED status, transfer status, and presence of cerebral complication; complex survey design and weighting were accounted for in analysis; age and CCI were modeled as restricted quadratic splines; an interaction term was used to obtain estimates across IV drug use status  ^b^ p-value comparing the combined effect of DU-IE and thoracic complications on patient outcomes  ^c^ Among patients who underwent the procedure only | | | |
