# Supplementary material for: Pulmonary complications observed in patients with infective endocarditis with and without injection drug use: An analysis of the National Inpatient Sample
Source: PLoS One. 2021 Sep 3;16(9):e0256757. doi: 10.1371/journal.pone.0256757 (PMC8415585; doi:10.1371/journal.pone.0256757)
Supplement: Attachment — Submitted filename: Endocarditis Manuscript_Response to Reviewers_PLOS_Final.docx [file pone.0256757.s002.docx]

January 28, 2021

Dear editor and reviewers,

We thank the academic editor and reviewers for taking the time to review our paper, and we appreciate their comments. We have attached the editor’s and reviewers’ comments and our responses below:

**Academic Editor Comments**

1. **Please ensure that your manuscript meets PLOS ONE's style requirements, including those for file naming.**

We have revised the manuscript to meet PLOS ONE’s style requirements.

2. **Thank you for providing the date(s) when patient medical information was initially recorded. Please also include the date(s) on which your research team accessed the databases/records to obtain the retrospective data used in your study.**

Our research team accessed the NIS database between July 2019 and November 2019 to obtain the retrospective data used in the study. This change is reflected in the “Methods and Materials” section of our paper.

3. **Please provide p-values for at least tables 2 and 3 in order to support your claims.**

P-values have been added to Tables 2 and 3.

4. **Thank you for stating the following in the Funding Section of your manuscript:**

**"AJS was funded by the National Institute on Drug Abuse (K23DA049946) and the National Institute of Allergy and Infectious Diseases (T32AI070114)."**

**We note that you have provided funding information that is not currently declared in your Funding Statement. However, funding information should not appear in the Acknowledgments section or other areas of your manuscript. We will only publish funding information present in the Funding Statement section of the online submission form.**

**Please remove any funding-related text from the manuscript and let us know how you would like to update your Funding Statement. Currently, your Funding Statement reads as follows:  "The author(s) received no specific funding for this work."**

We have removed any funding-related text from the manuscript. We wish to amend our current financial disclosure statement to state that “AJS was funded by the National Institute on Drug Abuse (K23DA049946) and the National Institute of Allergy and Infectious Diseases (T32AI070114). The funders had no role in study design, data collection and analysis, decision to publish, or preparation of the manuscript.”

5. **Please include captions for your Supporting Information files at the end of your manuscript, and update any in-text citations to match accordingly.**

Captions have been included for the Supporting Information files.

**Reviewer Comments #1**

1. **The site of affected valve and vegetation size could be helpful for detection and management of pulmonary complications and have predictor prognostic value**.

We agree that the site of the affected valve and vegetation size could be helpful for the detection and management of pulmonary complications and add prognostic value. However, the National Inpatient Sample database does not include valve location on non-surgical patients or vegetation size data. This is reflected in the “Limitation” section of the paper.

2. **Where are p-values for baseline data?**

We did not include p-values for baseline data because it is recommended by the American Statistical Association to exclude p-values on baseline characteristics. With large sample sizes, all baseline data would be statistically significant, and therefore, p-values would not be helpful with interpretation of the data.

We have provided two articles for your reference here:

Wasserstein RL, Lazar NA. The ASA Statement on p-Values: Context, Process, and Purpose. The American Statistician. 2016 Apr 2;70(2):129–33.

Wasserstein RL, Schirm AL, Lazar NA. Moving to a World Beyond “p < 0.05.” The American Statistician. 2019 Mar 29;73(sup1):1–19.

3. **Manuscript should follow the journal instruction regarding arrangement, tables and diagrams.**

The manuscript has been revised to follow journal instruction regarding arrangement, tables, and diagrams.

4. **Alphabetical and grammatical mistakes.**

Alphabetical and grammatical mistakes have been revised.

5. **Death was higher in non-pulmonary complication group 9% vs 8% of pulmonary complication group this is against data in line 278.**

Although the crude percentage of IE patients with non-pulmonary complications who died was greater than the percentage of IE patients with pulmonary complications who died, patients hospitalized with IE and pulmonary complications still had an increased odds of inpatient mortality (OR 1.81, 95% CI 1.39, 2.35), compared to those without pulmonary involvement after adjustment. This small observed reversed association in mortality is likely explained by differences in patient characteristics (i.e. confounding).

Reviewer Comments #2

1. **In Table 1, the rates of those using IV substances and those with HCV infection should be given according to the variables in the column.**

Table 1 has been revised. We have also added footnotes to clarify how IV drug use was assigned and analyzed in the table.

2. **The level of significance should be specified by statistically comparing the data in Table 1. In addition, it should be shown statistically whether there is any difference between the two groups, such as other comorbid diseases, central nervous system septic embolism, stroke, symptomatic embolism to other systems and organs, valvular heart disease, underlying chronic lung disease and immunosuppressive disease.**

As previously mentioned above, we did not include p-values for the baseline data in Table 1 due to the current recommendations by the American Statistical Association. Further, given that this is a relatively younger and ‘healthier’ patient population, we chose to use a composite score of the Charlson Comorbidity Index rather than including specific comorbidities due to the likely low prevalence of each individual comorbidity.

3. **In the comparison in which mortality was evaluated, were other causes that could affect mortality controlled?**

Yes, we controlled for several patient characteristics when assessing all-cause in-patient mortality. Specifically, we adjusted for age, sex, race/ethnicity, primary insurance, median income in the patient’s ZIP code, Charlson Comorbidity Index, IV drug use, hospital bed size, teaching status, region, ownership, elective admission, ED status, transfer status, and presence of cerebral complication. This is noted in our Methods section and included in the footnotes of Table 2 and 3. Although, it is likely that there may be some residual confounding (e.g. frailty) that we are unable to control and account for. We have added this to our Limitations section.

We did not and cannot assess specific causes of death in this dataset.

4. **Wasn't there any AIDS patient among 88,995 patients?**

Thank you for highlighting that HIV is a co-occurring concern, particularly in persons who inject drugs. In this study, we did not specifically examine HIV or AIDS as a comorbidity. AIDS is included in the Charlson Comorbidity Index (CCI), so the comorbidity conferred by AIDS is accounted for in that scoring. We opted not to specifically highlight the number of hospitalizations coded for AIDS, or other specific comorbidities, since this would introduce substantial complexity, given that many multisystem comorbidities may impact outcomes in patients with endocarditis or those undergoing surgery. We instead relied on the CCI as a summative measure.

5. **What was your purpose in calculating the CCI score? Did you not reach the necessary medical records to calculate this score? Would it be more appropriate to compare the QSofa and SOFA scores between groups instead?**

We opted to not highlight specific comorbidities given the likely low prevalence of these comorbidities in our patient population and the potential that many multisystem comorbidities may impact outcomes in patients with endocarditis or those undergoing surgery. We instead relied on the CCI as a summative measure. QSOFA and SOFA scores cannot be calculated using the data available in the National Inpatient Sample database.
