# Supplementary material for: Pulmonary complications observed in patients with infective endocarditis with and without injection drug use: An analysis of the National Inpatient Sample
Source: PLoS One. 2021 Sep 3;16(9):e0256757. doi: 10.1371/journal.pone.0256757 (PMC8415585; doi:10.1371/journal.pone.0256757)
Supplement: Attachment — Submitted filename: Endocarditis Manuscript_Response to Reviewers_PLOS_3.7.docx [file pone.0256757.s003.docx]

March 4, 2021

Dear editor and reviewers,

We thank the academic editor and reviewers for taking the time to re-review our paper, and we appreciate their comments. We have also added an additional author to our manuscript, Dr. Chris Agala. Our previous statistician and co-author Dr. Paula Strassle accepted a new position at the NIH; therefore, Dr. Agala assisted with the additional analyses we completed for this revision.

We have attached the reviewers’ comments and our responses below:

Reviewer Comments #1

1. **Unfortunately, the author did not address any of the previously mentioned recommendations with clear explanation other than no available data.**

We have included the previously requested baseline p-values in Table 1. Given the statistically significant demographic differences seen in Table 1 between the pulmonary complication and no pulmonary complication groups, we conducted secondary inferential analyses to gauge potential effects of a large sample size on the multivariate estimates of group comparisons. We assessed crude associations between outcomes and hospital regions as this was the only non-significant comparison in Table 1. The analysis showed some regional variations in all outcomes except heart valve surgery, even though the presence of pulmonary complications did not vary by hospital region (S3 Table). These results demonstrate that the differences seen in our outcomes of interest, such as length of stay, hospital charges, and discharge disposition (including inpatient mortality), are not only due to the differences seen in baseline characteristics of our study population.

As previously suggested, we have proofread the manuscript for any grammatical and spelling mistakes and have included that the reviewer’s suggested variables (i.e. site of affected valve and vegetation size) are not available to be collected and analyzed in the National Inpatient Sample database in our “Limitations” section.

**Table 1. Patient demographics and hospital characteristics, stratified by inpatient pulmonary complication**

|  | **Pulmonary Complication^a^**  **6,580 (7%)** | **No complication**  **82,415 (93%)** | **p-value** |
| --- | --- | --- | --- |
| **Age, years, median (IQR)** | 34 (27 – 46) | 66 (52 – 78) | <0.0001 |
| **Male, n (%)** | 3,145 (48) | 44,630 (54) | <0.0001 |
| **Race/ethnicity, n (%)** |  |  |  |
| Non-Hispanic White | 5,025 (81) | 57,115 (72) | <0.0001 |
| Non-Hispanic Black | 560 (9) | 10,725 (14) |  |
| Hispanic | 420 (7) | 7,090 (9) |  |
| Other^b^ | 195 (3) | 4,065 (5) |  |
| *Missing* | 380 | 3,420 |  |
| **Primary insurance, n (%)** |  |  |  |
| Medicare/Medicaid | 4,275 (66) | 63,330 (77) | <0.0001 |
| Private | 1,070 (17) | 13,990 (17) |  |
| Self-pay/other | 1,120 (17) | 4,690 (6) |  |
| **Median ZIP code income^c^, n (%)** |  |  |  |
| <$43,000 | 2,620 (41) | 26,735 (33) |  |
| $43,000-$53,999 | 1,725 (27) | 21,115 (26) | <0.0001 |
| $54,000-$70,999 | 1,230 (19) | 17,630 (22) |  |
| ≥$71,000 | 875 (13) | 15,205 (19) |  |
| **CCI, median (IQR)** | 0 (0 – 1) | 2 (0 – 3) | <0.0001 |
| **IV drug use, n (%)^d^** | 4,395 (67) | 11,095 (13) | <0.0001 |
| Opioid | 3165 (72) | 6670 (60) |  |
| Cocaine | 145 (3) | 855 (8) |  |
| Other drugs^e^ | 760 (18) | 2600 (23) |  |
| Hepatitis C only | 325 (7) | 970 (9) |  |
| **Hospital bed size, n (%)** |  |  |  |
| Small | 930 (14) | 13,625 (17) | 0.0030 |
| Medium | 1,625 (25) | 23,305 (28) |  |
| Large | 4,025 (61) | 45,485 (55) |  |
| **Hospital location/type, n (%)** |  |  |  |
| Rural, non-teaching | 375 (6) | 7,575 (9) | <0.0001 |
| Urban, non-teaching | 1,325 (20) | 20,195 (25) |  |
| Urban, teaching | 4,880 (74) | 54,645 (66) |  |
| **Hospital region, n (%)** |  |  |  |
| Northeast | 1,030 (16) | 15,650 (19) | 0.0750^f^ |
| Midwest | 1,325 (20) | 17,235 (21) |  |
| South | 3,015 (46) | 34,670 (42) |  |
| West | 1,210 (18) | 14,860 (18) |  |
| **Hospital ownership, n (%)** |  |  |  |
| Government, nonfederal | 1,000 (15) | 9,570 (12) | <0.0001 |
| Private, non-profit | 4,870 (74) | 59,370 (72) |  |
| Private, invest-own | 710 (11) | 13,475 (16) |  |
| **Elective admission, n (%)** | 375 (6) | 8,360 (10) | <0.0001 |
| **ED services, n (%)** | 4,170 (63) | 55,990 (68) | <0.0001 |
| **Admission source, n (%)** |  |  |  |
| Acute care transfer | 1,795 (28) | 11,680 (14) | <0.0001 |
| Other transfer | 220 (3) | 3,695 (5) |  |
| Non-transfer | 4,530 (69) | 66,720 (81) |  |
| **Cerebral complication, n (%)^f^** | 480 (7) | 8,780 (11) | <0.0001 |
| Abbreviations: ED, emergency department; CCI, Charlson comorbidity index  ^a^ Complications included pyothorax, lung abscess, and septic pulmonary embolism; patients were able to have more than one complication  ^b^ Other race includes Asian/Pacific Islander, Native American, and ‘Other’ as categorized by HCUP; races were collapsed due to small cell sizes  ^c^ Quartile classification of the estimated median household income of residents in the patient's ZIP Code  ^d^ Drug use was categorized as opioid use (with or without other drugs), cocaine (with or without other non-opioid drugs), other drugs (no opioid or cocaine), and Hepatitis C only  ^d^ Other drugs include benzodiazepines, amphetamines or stimulants, and hallucinogens.  ^e^ Includes cerebral abscess, hemorrhage, infarction, and ischemia  ^f^ Results from secondary analyses showed some regional variations in all outcomes except heart valve surgery, even though the presence of pulmonary complications did not vary by hospital region (S3 Table). | | | |

**S3 Table.** **Odds ratios and regression results for univariate model of outcomes and hospital region**

| **Outcomes by Hospital Region** | **OR (95% CI)** | **p-value** |
| --- | --- | --- |
| **Disposition and hospital region** |  |  |
| Routine (Northeast v. South) | 1.48 (1.31, 1.67) | <.0001 |
| Home health care (Northeast v. South) | 1.81 (1.55, 2.10) | <.0001 |
| Transferred (Northeast v. South) | 1.75 (1.57, 1.94) | <.0001 |
| Died (Northeast v. South) | 1.58 (1.35, 1.85) | <.0001 |
| Routine (Midwest v. South) | 1.14 (1.02, 1.29) | 0.0244 |
| Home health care (Midwest v. South) | 1.37 (1.18, 1.58) | <.0001 |
| Transferred (Midwest v. South) | 1.45 (1.31, 1.60) | <.0001 |
| Died (Midwest v. South) | 1.23 (1.05, 1.43) | 0.0092 |
| Routine (West v. South) | 0.85 (0.75, 0.96) | 0.0069 |
| Home health care (West v. South) | 1.05 (0.91, 1.23) | 0.4965 |
| Transferred (West v. South) | 0.93 (0.84, 1.03) | 0.1439 |
| Died (West v. South) | 1.02 (0.87, 1.19) | 0.7895 |
| **Thoracic procedure and hospital region** |  |  |
| Northeast v. South | 1.25 (1.03, 1.50) | 0.0219 |
| Midwest v. South | 1.10 (0.92, 1.30) | 0.3284 |
| West v. South | 0.95 (0.80, 1.19) | 0.532 |
| **Heart valve surgery and hospital region** |  |  |
| Northeast v. South | 1.13 (0.90, 1.41) | 0.2819 |
| Midwest v. South | 1.15 (0.84, 1.56) | 0.3859 |
| West v. South | 0.96 (0.76, 1.21) | 0.7137 |
| **Length of stay (days) and hospital region** |  | 0.0058 |
| Hospital charges (thousands) and hospital region |  | 0.0044 |
| Thoracic procedure (days) and hospital region |  | 0.0053 |
| Heart valve surgery (days) and hospital region |  | 0.0146 |
| Abbreviations: OR, odds ratio |  |  |

Reviewer Comments #2

1. **It would be much more useful and instructive if this study included the affected heart valve, culture results and causative microorganism. Author should put down these inadequacies in limitation part.**

We agree that the site of the affected valve, culture results, and causative microorganism would have been useful in our study analysis. However, the National Inpatient Sample database does not include valve location on non-surgical patients, culture results, or causative microorganism. This is now reflected in the “Limitations” section of the paper.
