# Supplementary material for: Pulmonary complications observed in patients with infective endocarditis with and without injection drug use: An analysis of the National Inpatient Sample
Source: PLoS One. 2021 Sep 3;16(9):e0256757. doi: 10.1371/journal.pone.0256757 (PMC8415585; doi:10.1371/journal.pone.0256757)
Supplement: Attachment — Submitted filename: Endocarditis Manuscript_Response to Reviewers_PLOS_3.25.docx [file pone.0256757.s004.docx]

April 4, 2021

Dear editor and reviewers,

We thank the academic editor and reviewers for taking the time to re-review our paper, and we appreciate their comments.

We have attached the reviewers’ comments and our responses below:

Reviewer Comments #3

1. **I suggest changing the aim and also the title of this paper. Most relevant conclusion of this paper is the high percentage of pulmonary complications observed in patients with endocarditis related to drug abuse. The greater impact on outcome of pulmonary complications in patients with endocarditis is expected and obvious and no data are available on major determinants of pulmonary complications.**

Thank you for your suggestions. We have changed the title of our paper to, “Pulmonary complications observed in patients with infective endocarditis with and without injection drug use: an analysis of the National Inpatient Sample”. However, we believe that the aims of our study were appropriate for our research question. Although, the greater impact on outcome of pulmonary complications in patients with IE may be expected, there is currently no literature with statistical data that supports this hypothesis. Our paper provides this evidence for both IE patients with and without drug use.

Reviewer Comments #4

1. **The background is not coherent and still does not demonstrate strong rationale supported by proper literature.**

We have revised the background with stronger rationale, including adding new references and figures.

2. **In the methodology, the authors should describe the inclusion and exclusion criteria in a clear manner. Moreover, better to define primary and secondary outcome of your search.**

All hospitalizations of adult (≥18 years old) patients diagnosed with IE were identified using ICD-10-CM codes and included in the study population. Patients discharged against medical advice or with unknown disposition were excluded from the study population since their course of care and clinical outcomes could not be reliably assessed.

The primary outcome of interest was inpatient mortality in IE-PC patients (with and without drug use). The secondary outcomes of interest included undergoing a thoracic and/or heart valve procedure, discharge disposition, LOS, total hospital charges, and time to thoracic or heart valve procedure (among those who underwent procedure only).

3. **The authors have selected the year of 2016 which was the first year to use ICD 10. This may mean higher human error.**

Yes, we agree with your comment and have mentioned the potential for coding errors with using an administrative discharge database in the “Limitations” section. However, this missing data is expected to bias results towards the null (i.e. underestimate the true magnitude of our estimates).

4. **No details on survival or follow up. Data were collected from administrative electronic archive and lack important clinical data.**

We recognize that this is a limitation of a large administrative database. The National Inpatient Sample database only includes data during a patient’s hospitalization. Therefore, details on survival or follow-up after discharge are not captured in the database. This now reflected in the “Limitations” section of our manuscript. However, this is an excellent topic for future study using multi-institutional data or the STS National Database that allows for more granular data collection.

5. **The large sample size from electronic record has its own limitations. The authors should defend more regarding this point.**

We agree that a large sample size has its own limitations. Of note, it can be likely to find a difference by chance. However, for that reason, we performed a secondary inferential analysis to assess the crude association between outcomes and hospital regions. As mentioned in the “Methodology” section, the analysis was performed to gauge the potential effect of a large sample size. The analysis confirmed that our findings were not by chance but were in fact real effects as seen in the “Results” section and Supplemental Table 3. We have also adhered to the guidelines suggested by JAMA Surgery for improving the quality of database research.

Haider AH, Bilimoria KY, Kibbe MR. A Checklist to Elevate the Science of Surgical Database Research. *JAMA Surg*. 2018;153(6):505. doi:10.1001/jamasurg.2018.0628

6. **Search and analyze important data like hospital readmission within 90 days after discharge.**

While we agree that hospital readmission data is important, the National Inpatient Sample database does not include hospital readmission data. This is now reflected in the “Limitations” section of our manuscript. This is also an excellent topic for future study using multi-institutional data or the STS National Database that allows for more granular data collection.
