# Supplementary material for: Pulmonary complications observed in patients with infective endocarditis with and without injection drug use: An analysis of the National Inpatient Sample
Source: PLoS One. 2021 Sep 3;16(9):e0256757. doi: 10.1371/journal.pone.0256757 (PMC8415585; doi:10.1371/journal.pone.0256757)
Supplement: Attachment — Submitted filename: Endocarditis Manuscript_Response to Reviewers_PLOS_5.2.docx [file pone.0256757.s005.docx]

May 11, 2021

Dear editor and reviewers,

We thank the academic editor and reviewers for taking the time to re-review our paper, and we appreciate their comments.

We have attached the comments and our responses below:

Editor Comments:

1. **Please review your reference list to ensure that it is complete and correct. If you have cited papers that have been retracted, please include the rationale for doing so in the manuscript text, or remove these references and replace them with relevant current references. Any changes to the reference list should be mentioned in the rebuttal letter that accompanies your revised manuscript. If you need to cite a retracted article, indicate the article’s retracted status in the References list and also include a citation and full reference for the retraction notice.**

The reference list has been reviewed for accuracy. We did not make any changes to the reference list or cite any retracted articles.

Reviewer #3 Comments:

1. **My major comment was not addressed. Patients with IE and pulmonary complications have worse outcomes, and this is obvious, however, more interesting, major determinants of pulmonary complications in patients with IE were not studied.**

We have added additional analyses examining the predictors of pulmonary complications in patients with IE. It was confirmed in our analysis that DU-IE patients had the highest odds of pulmonary complications. Other predictors of pulmonary complications included: elective admission to the hospital, acute transfer status, self-paying status, and treatment at an urban institution. Predictors associated with decreased odds of pulmonary complications included older age, higher CCI, NH Black and Hispanic patients, and the presence of cerebral complications. This is now reflected in our Aims, Methods, Results, and Discussion of our paper.
