# Supplementary material for: Pulmonary complications observed in patients with infective endocarditis with and without injection drug use: An analysis of the National Inpatient Sample
Source: PLoS One. 2021 Sep 3;16(9):e0256757. doi: 10.1371/journal.pone.0256757 (PMC8415585; doi:10.1371/journal.pone.0256757)
Supplement: Attachment — Submitted filename: Endocarditis_Response to Reviewers_6.8.docx [file pone.0256757.s006.docx]

June 8, 2021

Dear editor and reviewers,

We thank the academic editor and reviewers for taking the time to re-review our paper, and we appreciate their comments.

We have attached the comments and our responses below:

Reviewer #6:

1. **According to the Table 3, it seems that the mortality is significantly higher in the patients without pulmonary complication (8% vs 9%, OR 1.81, p<0.0001). This result is completely opposite to the interpretation in the manuscript, so the authors need to clarify this point.**

Although the crude percentage of IE patients with non-pulmonary complications who died was greater than the percentage of IE patients with pulmonary complications who died, patients hospitalized with IE and pulmonary complications still had an increased odds of inpatient mortality (OR 1.81, 95% CI 1.39, 2.35), compared to those without pulmonary involvement after adjustment. This small observed reversed association in mortality is likely explained by differences in patient characteristics (i.e. confounding). This point has been clarified in the Results and Limitations section.

2. **The authors divided the patients into groups named “Pulmonary complication” and “No complication” in Table 1, 2 and 3. However, the reviewer thinks “No complication” is a misleading term. “No pulmonary complication” or “Without pulmonary complication” should be appropriate.**

Thank you for the suggestion. We have changed the term to “No Pulmonary Complication” in Table 1, 2, and Supplemental Table 3.  **3. There are significant overlap in the contents between Table 1 and Table 2. The reviewer thinks it is better to put aside one of which to the supplementary data.**

Table 2 have been moved to the supplementary data as S3 Table.  **4. Table 4 only shows the patients outcomes. The comparison of patient demographics and characteristics according to IV drug use should be also presented in a Table format.**

We have added a Table 3 that includes patient demographics and hospital characteristics among patients with and without drug use.

**5. Because only 7% of the whole population has pulmonary complication, the absolute number of drug users is higher in patients without pulmonary complications. Therefore, the number and percentage of each group should be listed in the first column of Table 4 (as in Table 1 and Table 2).**

We were a bit confused on this suggestion but tried our best to interpret your suggestion. We have added the total number and percentage of DU-IE and non-DU-IE patients to the first row in Table 4. We will be happy to revise further if this was not your intended suggestion.
