# Supplementary material for: Pulmonary complications observed in patients with infective endocarditis with and without injection drug use: An analysis of the National Inpatient Sample
Source: PLoS One. 2021 Sep 3;16(9):e0256757. doi: 10.1371/journal.pone.0256757 (PMC8415585; doi:10.1371/journal.pone.0256757)
Supplement: Attachment — Submitted filename: Endocarditis Manuscript_Response to Reviewers_PLOS_7.26.docx [file pone.0256757.s007.docx]

July 25, 2021

Dear editor and reviewers,

We thank the academic editor and reviewers for taking the time to re-review our paper, and we appreciate their comments.

We have attached the comments and our responses below:

**Reviewer #6:**

1. **The authors addressed the concerns raised by the reviewer except for the major problem #1; the authors provide an explanation for the reversed association in mortality as a response to the reviewer, but it is not reflected in the manuscript, although they stated that “This point has been clarified in the Results and Limitations section”.

   The fact that "patients with pulmonary complications have a statistically significantly lower crude mortality rate, but when adjusted for factors such as age and gender, the results are reversed" is extremely important and should not be dismissed out of hand. Looking at Table 1, patients with pulmonary complications are younger and have lower rates of cerebrovascular complications, so it is unlikely that the odds ratio would be reversed to 1.8 even after adjustment for clinical factors. It is necessary to make an effort to identify which confounders are influencing the results, and it is the minimum obligation of scientists to state this in discussions and limitations. Otherwise, it is suspected that the method of analysis and interpretation may have been incorrect.**

As previously mentioned, although the crude percentage of IE patients without pulmonary complications who died was greater than the percentage of IE patients with pulmonary complications who died, patients hospitalized with IE and pulmonary complications still had an increased odds of inpatient mortality (OR 1.81, 95% CI 1.39, 2.35), compared to those without pulmonary involvement after adjustment. In fact, our additional analysis shows that the odds ratio for mortality compared to routine discharge disposition was lower among patients with no pulmonary complications compared to those with pulmonary complications (OR: 0.878; 95% CI: 0.703 ,1.095), in line with crude frequencies of 8% (pulmonary omplications) vs. 9% (without pulmonary complications) in Table 2. This small observed reversed association in mortality is likely explained by differences in patient and clinical characteristics which were included in the adjusted model and produced the reported adjusted odds ratio of 1.81. One potential confounding factor that could explain this finding includes:

1) The Charleson Comorbidity Index (CCI) is a measure of comorbidities at the time of admission and includes diseases such as myocardial infarction, congestive heart failure, peripheral vascular disease, cerebrovascular disease, dementia, chronic pulmonary disease, rheumatologic disease, peptic ulcer disease, liver disease, diabetes mellitus without complications, diabetes complications, hemiplegia/paraplegia, renal disease, metastatic tumor, HIV/AIDS. In Table 1, we observe that those with pulmonary complications had a median CCI of 0 (IQR:0-1), suggesting half of them had no comorbidity, compared to a median CCI of 2 (IQR:0-3) among those with no pulmonary complications. Those with a higher number of comorbidities also tend to have higher mortality rates. Therefore, patients were likely to die of other causes as well. Additional analysis shows that 14% of all patients who died had a CCI value of 0, the rest had a higher CCI value. Overall, this analysis does not suggest that having pulmonary complications was the cause of death as cause of death is not reported in NIS data. It is also possible that some of this difference could be accounted for by omitted variables, which were also unavailable in the database—one of the limitations of the NIS database (please see limitations of NIS in McGinigle et al. 2021 (https://www.sciencedirect.com/science/article/pii/S0741521420325064?via%3Dihub))

While we did account for differences in demographics, some clinical and hospital characteristics, and comorbidities between groups, including CCI, we cannot account for all the differences in Table 1, and it is likely that some residual confounding that we are unable to account for still exists. Please see this text in the Discussion section on page 16, and in Limitations section on page 17, with added relevant citations.
